# Presence and Persistence of *ESKAPEE* Bacteria before and after Hospital Wastewater Treatment

**DOI:** 10.3390/microorganisms12061231

**Published:** 2024-06-19

**Authors:** Miguel Galarde-López, Maria Elena Velazquez-Meza, Elizabeth Ernestina Godoy-Lozano, Berta Alicia Carrillo-Quiroz, Patricia Cornejo-Juárez, Alejandro Sassoé-González, Alfredo Ponce-de-León, Pedro Saturno-Hernández, Celia Mercedes Alpuche-Aranda

**Affiliations:** 1 Centro de Investigación Sobre Enfermedades Infecciosas, Instituto Nacional de Salud Pública, Morelos 62100, Mexico; miguel.galarde@insp.edu.mx (M.G.-L.); elizabeth.godoy@insp.mx (E.E.G.-L.); berta.carrillo@insp.mx (B.A.C.-Q.); 2 Departamento de Infectología, Instituto Nacional de Cancerología, Tlalpan, Mexico City 14080, Mexico; patcornejo@yahoo.com; 3 Unidad de Inteligencia Epidemiológica, Hospital Regional de Alta Especialidad de Ixtapaluca, Ixtapaluca 56530, Mexico; sassoe777@hotmail.com; 4 Laboratorio Nacional de Máxima Seguridad para el Estudio de Tuberculosis y Enfermedades Emergentes, Instituto Nacional de Ciencias Médicas y Nutrición “Salvador Zubirán”, Mexico City 14080, Mexico; alf.poncedeleon@gmail.com; 5 Centro de Investigación en Evaluación de Encuestas, Instituto Nacional de Salud Pública, Morelos 62100, Mexico; pedro.saturno@insp.mx

**Keywords:** antimicrobial resistance, *ESKAPEE*, metagenomic, resistome, wastewater, public health

## Abstract

The metagenomic surveillance of antimicrobial resistance in wastewater has been suggested as a methodological tool to characterize the distribution, status, and trends of antibiotic-resistant bacteria. In this study, a cross-sectional collection of samples of hospital-associated raw and treated wastewater were obtained from February to March 2020. Shotgun metagenomic sequencing and bioinformatic analysis were performed to characterize bacterial abundance and antimicrobial resistance gene analysis. The main bacterial phyla found in all the samples were as follows: *Proteobacteria*, *Bacteroides*, *Firmicutes*, and *Actinobacteria*. At the species level, *ESKAPEE* bacteria such as *E. coli* relative abundance decreased between raw and treated wastewater, but *S. aureus*, *A. baumannii*, and *P. aeruginosa* increased, as did the persistence of *K. pneumoniae* in both raw and treated wastewater. A total of 172 different ARGs were detected; *bla*_OXA_, *bla*_VEB_, *bla*_KPC_, *bla*_GES_, *mphE*, *mef*, *erm*, *msrE*, *AAC(6′)*, *ant(3″)*, *aadS*, *lnu*, *PBP-2*, *dfrA*, *vanA-G*, *tet*, and *sul* were found at the highest abundance and persistence. This study demonstrates the ability of *ESKAPEE* bacteria to survive tertiary treatment processes of hospital wastewater, as well as the persistence of clinically important antimicrobial resistance genes that are spreading in the environment.

## 1. Introduction

The epidemiological surveillance of antimicrobial resistance (AMR) has been suggested as an essential methodological tool to observe the distribution, status, and trends of antibiotic resistant bacteria [1]. The surveillance of clinically important bacteria in the wastewater environment has gained relevance in hospital settings, community settings, and animal production, mainly because various components such as biomarkers of the microorganisms present, detergents, trace antibiotics, and other substances converge in wastewater systems [2]. Hospital wastewaters are considered the main environment that may contain clinically important antimicrobial-resistant pathogens due to selection pressure for the horizontal gene transfer of AMR [2,3,4]. 

The detection of bacterial communities, their resistance genes (ARGs), and/or mobile genetic elements (MGEs) in hospital wastewater systems can serve as an early warning tool for the potential spread of AMR to the environment [2,5,6,7].

Among the list of critically important bacteria described by the World Health Organization (WHO) [8] are the *ESKAPEE* group: *Enterococcus faecium*, *Staphylococcus aureus*, *Klebsiella pneumoniae*, *Acinetobacter baumannii*, *Pseudomonas aeruginosa*, *Enterobacter cloacae*, and *Escherichia coli. ESKAPEE* bacteria are rapidly acquiring resistance to antibiotics, so their dissemination in the environment through hospital wastewater may pose a serious problem [9,10,11]. Wastewater treatment plants (WWTPs) play a key role in decreasing bacterial abundance because they have been well-described, as well as hotspots that favor AMR through the transfer of genetic material [12,13].

Although WWTPs reduce the abundance of bacteria released into the environment, current wastewater treatment processes do not remove all bacteria, antibiotic-resistant bacteria with their ARGs, and antibiotic residues. Some studies have demonstrated the importance of *ESKAPEE* group bacteria in hospital, community, or livestock wastewater as important sources of environmental epidemic pathogens [14]. Because hospital wastewater contributes to the burden of multidrug resistance, WWTP may facilitate the presence, persistence, and evolution of *ESKAPEE* group bacteria [15,16].

The use of metagenomic studies in specific environments such as wastewater has provided insight into the profile of bacterial communities through phylogenetic analyses and their resistome [17,18], which has provided advantages over conventional microbiology based on bacterial cultures with some selection biases. Furthermore, metagenomic studies of AMR bacteria have contributed significantly to existing antimicrobial resistance databases [19,20,21].

The AMR surveillance of *ESKAPEE* bacteria globally in the hospital wastewater setting is in line with the WHO Global Action Plan on Antimicrobial Resistance with the aim of “strengthening the science and knowledge base through surveillance and research” [22]. Therefore, the aim of this study was to describe the presence and persistence of *ESKAPEE* group bacteria and antimicrobial resistance genes in raw and treated wastewater from two tertiary-level hospitals in Mexico.

## 2. Materials and Methods

### 2.1. Study Design and Sample Collection

This descriptive cross-sectional study was conducted from February to March 2020. Three samples of raw (*n* = 3) and treated (*n* = 3) wastewater were collected in the WWTP of the Hospital Regional de Alta Especialidad de Ixtapaluca (HRAEI) with a 19.19116 N latitude and 98.51187 W longitude, and two samples of raw (*n* = 2) and treated (*n* = 2) wastewater were obtained from the WWTP of the Instituto Nacional de Cancerología (INCAN) with a 19.17128 N latitude and 99.06314 W longitude, both in central Mexico. One liter of each wastewater sample (raw and treated) was collected using the single grab technique in sterile containers, with an interval of one week between each sample collection. The samples were transported to the laboratory at 4 °C within less than two hours of collection [9,10].

### 2.2. Wastewater Treatment Plant Characteristics

The layout of both WWTPs begins with a pretreatment process using a grate that eliminates large materials. Then, the water flow is dosed to an aeration process mediated by regulating valves in different chambers. The treatment process continues with a compound sedimentation phase, precipitating the sludge. Tertiary treatment includes calcium hypochlorite tablets, granular activated carbon filters, and ultraviolet light (UVL) in the effluent.

### 2.3. Sample Processing

Aliquots of 100 mL of raw wastewater and 200 mL of treated wastewater were obtained and centrifuged at 5000× *g* for 20 min at 4 °C. The supernatant was decanted and one milliliter of EC lysis solution (1M Tris pH 8.0, EDTA, sodium deoxycholate, N-lauryl sarcosyl, RNAase, lysozyme and lysostaphin) was added to the pellet and incubated at 37 °C for four hours. Then, the ESP solution (EDTA + N-lauryl sarcosyl + proteinase K) was added and incubated at 50 °C overnight [23]. The samples were purified with the Wizard^®^ Genomic DNA Purification Kit (PROMEGA Corp., Madison, WI, USA) according to the manufacturer’s instructions. DNA was quantified by fluorometry using the Qubit 4 Fluorometer (Thermo Fisher Scientific, Waltham, MA, USA). The DNA samples were stored at −20 °C until sequencing.

### 2.4. Sequencing and Bioinformatics Analysis

The ten samples were sequenced by Illumina HiSeq (Illumina, Inc., San Diego, CA, USA), with 2 × 150 configuration (21.4 GB). Libraries were performed using the Nextera DNA protocol. The reads are available from NCBI SRA under BioProject ID **PRJNA1010860**.

Adapters were removed for raw reads and a Q > 20 was considered with Trimmomatic [24]. Quality control statistics were performed with FastQC [25]. Metagenome assemblies were performed with IDBA-UD (v1.1) [26,27]. Mapping statistics were performed with Bowtie 2 [28] and can be reviewed in Supplementary Material Table S1. For functional annotation, the Trinotate (v3.0.1) pipeline was employed [29,30]. Gene abundance was estimated as coverage by mapping reads to contigs using BWA (v0.7.12-r1039) and the coverBed function of bedtools (v2.25.0) [31,32].

MetaPhlAn (v4.0) was used for taxonomic profiling [33,34]. Differential abundance analysis between conditions at the species level was performed with the MetagenomeSeq R library [35]. Alpha and beta diversity analyses were performed with the vegan (v2.4-6) and phyloseq libraries in [36,37]. The distance matrix for beta diversity was performed with the Bray–Curtis index at the species level. Comparisons between groups were determined with an analysis of similarity (ANOSIM) [37,38]. The base 2 logarithm of the relationship between the two expression values was taken: LFC = log2 (A/B), where A and B represent the relative abundance levels of each genus in different wastewater conditions. Abundance plots and histograms were performed with the ggplot2 and ggpubr library of R [36,39,40].

### 2.5. Analysis of Antimicrobial Resistance Genes

The ARGs and resistome of the samples were determined using contigs of lengths of >150 bp using ABRicate (v1.0.1) [41], with the Comprehensive Antibiotic Resistance Database (CARD) (v3.2.9) [42] and PlasmidFinder (v2.0) [43]. The identified ARGs were grouped into classes according to the type of antibiotic to which they confer resistance, and quantified by absolute abundance, relative abundance, and normalized abundance expressed as a percentage. The virulence factor database (VFDB) (as of October 2023) and the *E. coli* O-groups and H-types database (EcOH) (as of October 2023) were used for virulence gene detection [44,45].

## 3. Results

### 3.1. Bacterial Composition of the Wastewater

**Figure 1:**
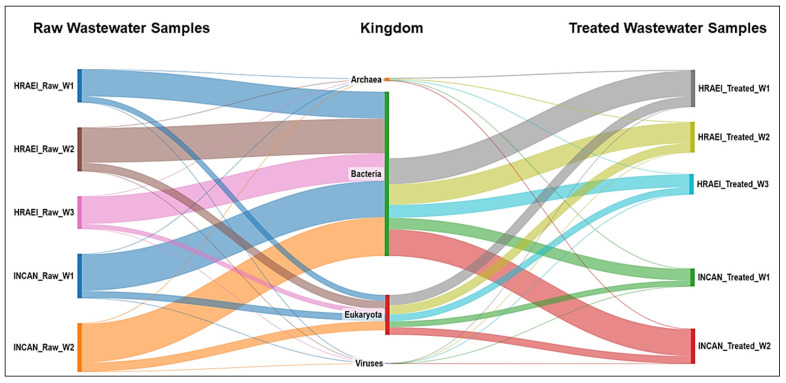
Bacterial community composition in hospital wastewater samples. Taxonomic annotation at the kingdom level.

The average abundance relative to the bacterial Kingdom was 81.2 ± 1.9% in the raw wastewater, while in the treated wastewater, it was 67.8 ± 5.1% for both hospitals (Figure 1), which represented an average decrease of 12.8% (14.7% for HRAEI and 11.1% for INCAN).

**Figure 2:**
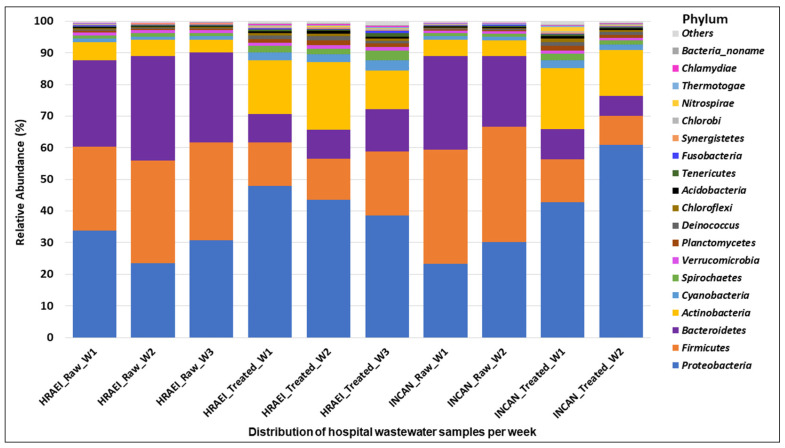
Bacterial community composition in hospital wastewater samples. Relative abundance at the phylum level.

The raw and treated wastewater samples presented a homogeneous pattern, finding 60 classes in 36 phyla of the bacterial kingdom. The main bacterial phyla found in raw wastewater samples above 1% relative abundance were *Firmicutes* (32 ± 4.1%), *Proteobacteria* (28 ± 4.7%), *Bacteroidetes* (28 ± 3.9%), and *Actinobacteria* (5 ± 0.6%), while in the treated wastewater samples, they were *Proteobacteria* (46 ± 8.5%), *Firmicutes* (13 ± 3.9%), *Bacteroidetes* (9 ± 2.5%), and *Actinobacteria* (16 ± 3.6%) (Figure 2).

**Figure 3:**
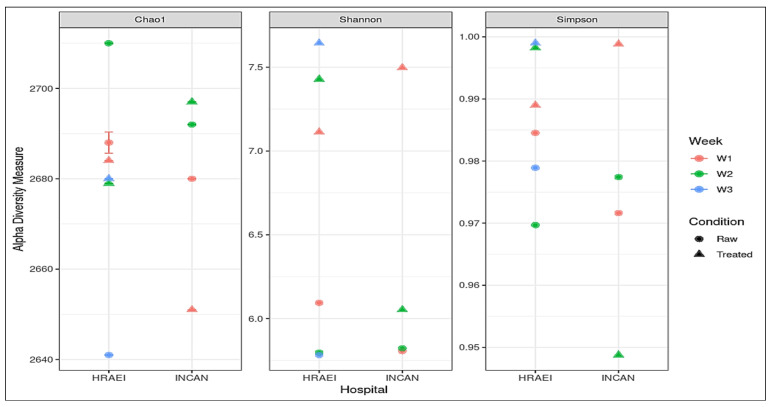
Alpha diversity indexes of each treatment at the species level, Chao1 diversity index, Shannon’s diversity index, and Simpson diversity index.

From the taxonomic-level species annotations, alpha diversity was determined with a *p*-value < 0.05 using Shannon’s index, where a greater richness of bacterial species was observed in the treated wastewater samples. The average observed richness of the wastewater samples was 2680 ± 20 unique species, and the average Chao1 index was 2680 ± 20. The bacterial diversity for all the samples was calculated using the Shannon index value, where we found values within the range of 5.78 to 7.64. The most diverse bacterial communities were those of the raw compared to treated wastewater samples, with species dominance (Simpson’s alpha diversity index), while the Chao1 diversity index showed similar richness between treatments with no significant differences detected (Figure 3).

**Figure 4:**
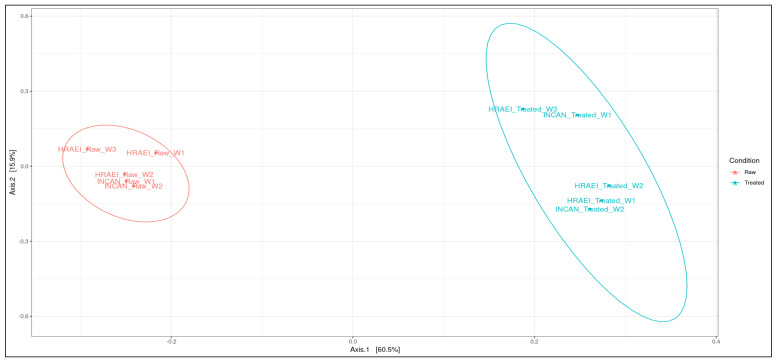
Analysis of similarity (ANOSIM). Principal coordinate analysis between treatments using the Bray–Curtis distance matrix at the species level.

Principal coordinate analysis (PCoA) was performed at the species level to determine the type of wastewater condition (raw and treated), the relationship between samples at the species level, and the time of sampling. In this analysis, it was observed that the wastewater samples from both hospitals were grouped according to wastewater condition (raw and treated), suggesting compositional differences between the two types of waters analyzed. Between sampling time intervals in treated wastewater, the differences in bacterial species composition were observed (*p*-value < 0.05). In the comparison of the wastewater samples, principal components 1 and 2 explained 76.4% of the variation after the processes in the WWTPs (Figure 4).

### 3.2. Differential Abundance of ESKAPEE Group Genera in Wastewater

The presence of 1004 bacterial genera was detected in the wastewater samples analyzed. The results of the average relative abundances for the bacterial genera of the *ESKAPEE* group from both hospitals were as follows: *Enterococcus* spp. 0.4 ± 0.1% in the raw wastewater and 0.5 ± 0.2% in the treated wastewater, *Staphylococcus* spp. 0.2 ± 0.1% and 0.3 ± 0.2%, respectively, *Klebsiella* spp. 0.4 ± 0.1% in the raw wastewater and 0.2 + 0.1% in the treated wastewater, *Acinetobacter* spp. 1.0 ± 0.5% and 1.9 ± 2.9%, respectively, *Pseudomonas* spp. 1.1 ± 0.2% in the raw wastewater and 7.1 ± 9.3 in the treated wastewater, *Enterobacter* spp. 0.3 ± 0.1% in the raw wastewater and 0.2 ± 0.1% in the treated wastewater, and *Escherichia* spp. 1.3 ± 0.0% and 0.2 ± 0.1%, respectively.

**Figure 5:**
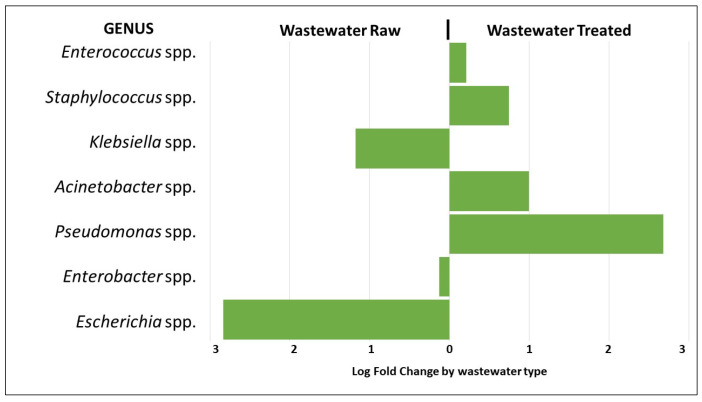
Analysis of the differential abundance of the genera of the *ESKAPEE* group by wastewater type.

The analysis of differential abundance of bacterial genera belonging to the *ESKAPEE* group showed approximately a three-fold greater abundance of *Pseudomonas* spp. in the treated wastewater compared to the raw wastewater. *Staphylococcus* spp. and *Acinetobacter* spp. showed approximately a one-fold greater abundance, respectively. On the other hand, the abundance of *Escherichia* spp. was approximately three-fold greater in the raw wastewater compared to the treated, and for *Klebsiella* spp., the abundance was approximately one-fold greater, respectively (*p* < 0.01) (Figure 5).

**Figure 6:**
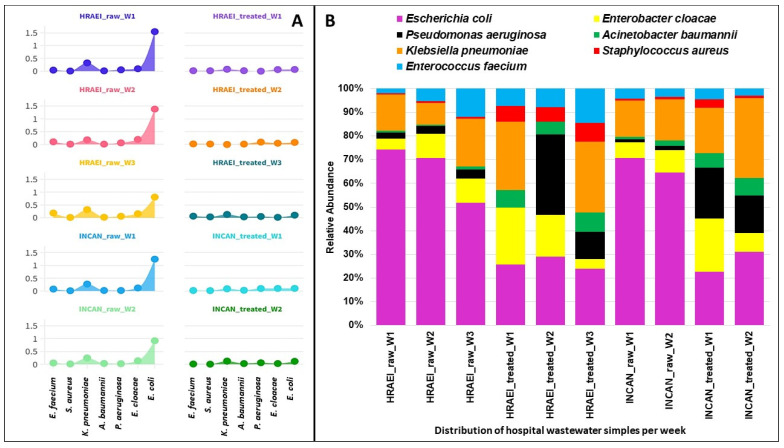
Relative abundance of *ESKAPEE* group bacteria at the species level. (**A**) Relative abundance (log) in each hospital wastewater sample before and after the wastewater treatment plant. (**B**) Relative abundance of *ESKAPEE* group bacteria.

The presence of 2837 bacterial species was detected in the wastewater samples analyzed. The results of the average relative abundances for the bacterial species of the *ESKAPEE* group from both hospitals were as follows: *Enterococcus faecium* 0.09 ± 0.05% in the raw wastewater and 0.02 ± 0.01% in the treated wastewater, *Staphylococcus aureus* 0.01 ± 0.01% and 0.01 ± 0.01%, respectively, *Klebsiella pneumoniae* 0.26 ± 0.05% in the raw wastewater and 0.08 ± 0.05% in the treated wastewater, *Acinetobacter baumannii* 0.02 ± 0.01% and 0.02 ± 0.01%, respectively, *Pseudomonas aeruginosa* 0.04 ± 0.02% in the raw wastewater and 0.05 ± 0.03% in the treated wastewater, *Enterobacter cloacae* 0.14 ± 0.03% in the raw wastewater and 0.05 ± 0.03% in the treated wastewater, and *Escherichia coli* 1.17 ± 0.31% and 0.09 ± 0.01%, respectively. A decrease in the relative abundance of *E. coli* was observed between raw and treated wastewater, while for *S. aureus*, *A. baumannii*, and *P. aeruginosa*, this abundance increased (*p* < 0.05). For *K. pneumoniae*, this abundance was similar under both conditions (Figure 6).

**Figure 7:**
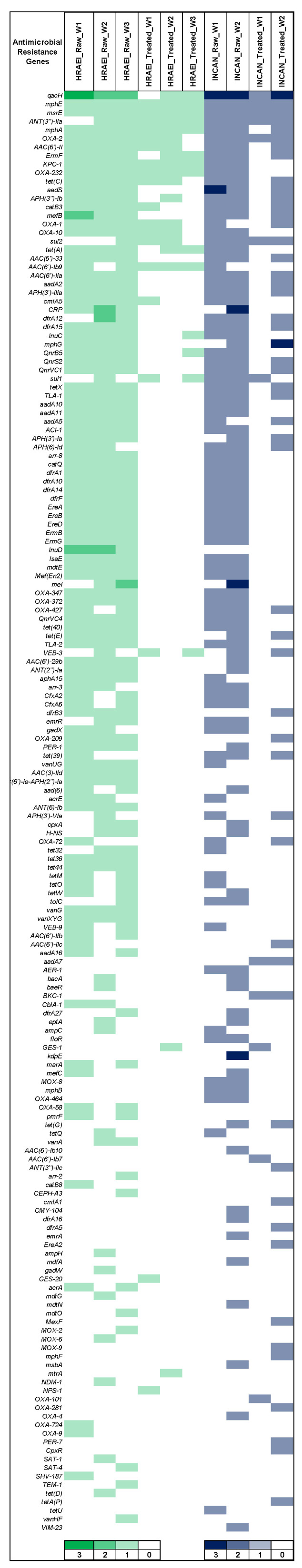
Relative abundance of antibiotic resistance genes by CARD. Green: HRAEI. Blue: INCAN.

The CARD database was used to determine the presence and identity of ARGs in each sample at various times, for the comparative analysis of the presence, relative abundance, and persistence of ARGs (CARD database). ARGs (172) were detected in both hospitals’ raw and treated wastewater samples. In raw wastewater samples from HRAEI, 102 + 2.8 ARGs and 17.3 + 2.8 ARGs were detected in treated wastewater. On the contrary, in the INCAN raw wastewater samples, 92 + 9.8 ARGs and 32 + 28.2 ARGs were identified in the treated wastewater. The antimicrobial resistance genes *bla*_OXA_, *bla*_VEB_, *bla*_KPC_, *bla*_GES_, *mphE*, *mef*, *erm*, *msrE*, *AAC(6′)*, *ant(3″)*, *aadS*, *lnu*, *PBP-2*, *dfrA*, *vanA-G*, *tet*, and *sul* had the highest abundance and persistence. These genes encode resistance for *β*-lactam, macrolides, tetracyclines, sulfonamides, aminoglycosides, lincosamide, and glycopeptides. Other ARGs such as *bla*_NDM_, *bla*_TEM_, and *acrA* genes were found at a lower abundance (Figure 7) (Supplementary Material Table S2).

PlasmidFinder analysis of raw and treated wastewater samples detected the persistence of plasmids ColKP3-1 carrying the ARGs *bla*_OXA-131_ and *bla*_OXA-232_; IncQ2_1 carrying the ARGs *sul2*, *strAB*, and *tetA*; ColRNAI_1 carrying *fosA*; and Col440I_1 and Col440II_1 carrying the ARGs *qnrB*, *cmlA1* and *fosA7*, mainly Enterobacterial. The main virulence factor genes persistent in the raw and treated wastewater samples were *icmJ* associated with endonucleases from *Pseudomonas* spp., *htpB* associated with adhesion in different enterobacteria, and *fliG* associated with adhesion and motility in several enterobacteria (Supplementary Material Table S2).

**Figure 8:**
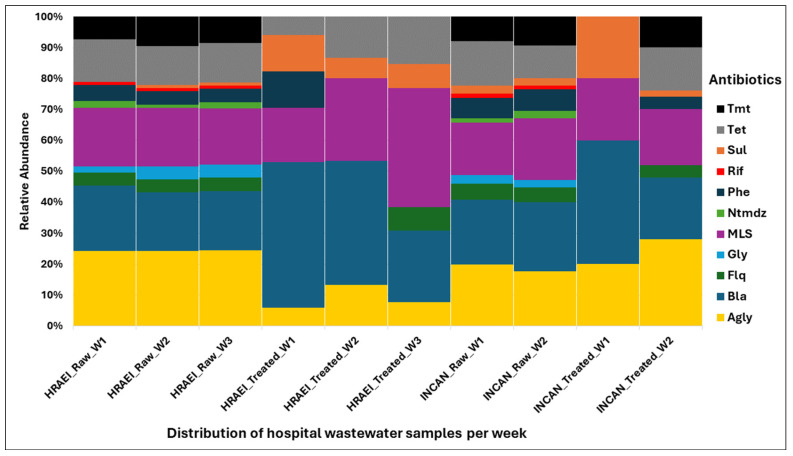
Relative abundance of resistance genes encoding antibiotic classes in wastewater samples. Drug class: Agly (aminoglycoside), Bla (betalactam), Flq (fluoroquinolana), Gly (glycopeptides), MLS (macrolides–lincosamides–streptogramines), Ntmdz (nitroimidazole), Phe (phenicol), Rif (rifampicin), Sul (sulfonamides), Tet (tetracycline), and Tmt (trimethoprim).

Overall, the ARGs present in each of the raw and treated wastewater samples were grouped in relation to the major antibiotic classes: aminoglycosides (AGly), β-lactams (Bla), fluoroquinolones (Flq), glycopeptides (Gly), macrolides–lincosamides–streptogramines (MLS), nitroimidazole (Ntmdz), phenicol (Phe), rifampicin (Rif), sulfonamides (Sul), tetracycline (Tet), and trimethoprim (Tmt). The highest abundance and persistence of resistance genes were those of aminoglycosides, β-lactams, and MLS compared to the other antibiotic classes detected (Figure 8).

## 4. Discussion

Wastewater is a vector that transports bacteria and resistance genes from the clinical setting to the environment, so it is essential to adequately treat this water [1]. It is important to evaluate these systems by sequencing techniques to establish the presence and abundance of bacteria and resistance genes in different settings [2,46,47]. This study, conducted in two hospital WWTP environments, showed that these WWTPs reduced the relative abundance of bacteria in treated wastewater by 13% compared to raw wastewater (Figure 1). This result was like that found in other studies conducted in the Netherlands [48], Germany [49], and China [50], in which WWTP processes reduced the relative abundance of pathogenic bacteria.

The microbial composition of the raw wastewater samples analyzed at the phyla level in our study was dominated by *Firmicutes* (32 ± 4.1%), *Proteobacteria* (28 ± 4.7%), *Bacteroidetes* (28 ± 3.9%), and *Actinobacteria* (5 ± 0.6%), while in the treated wastewater samples, it was *Proteobacteria* (46 ± 8.5%), *Firmicutes* (13 ± 3.9%), *Bacteroidetes* (9 ± 2.5%), and *Actinobacteria* (16 ± 3.6%). Numberger et al. also reported the predominance of Firmicutes, *Proteobacteria*, *Bacteroidetes*, and *Actinobacteria*, analyzing a hospital WWTP in four times of the year [49]. However, their results on the relative abundance of these phyla were different from those found in our study, where an average relative abundance was observed for *Firmicutes* (52.2 ± 4.4), *Proteobacteria* (37.8 ± 4.7%), *Bacteroidetes* (4.9 ± 1.9%), and *Actinobacteria* (2.2 ± 0.2%) [48]. The increase in *Proteobacteria* and *Actinobacteria* in treated wastewater samples observed in our results could be related to the type of samples associated with human biomes, as previously reported [51].

Particularly, the phyla of *Proteobacteria* and *Firmicutes*, which include the genera of the *ESKAPEE* group, have been referred to as the most abundant [51,52,53,54]. Our results showed that *Escherichia* spp., *Klebsiella* spp., and *Enterobacter* spp. were the predominant genera in the raw wastewater, while *Enterococcus* spp., *Staphylococcus* spp., *Acinetobacter* spp., and *Pseudomonas* spp. were found in the treated wastewater. In our study, we observed that *Escherichia* spp. and *Klebsiella* spp. were significantly reduced (<99% and <50%) in the WWTP. A similar behavior was observed by Verburg I. et al. [48,55], where the reduction in these genera was <90%. In our study, it was also observed that the relative abundance of *Acinetobacter* spp. and *Pseudomonas* spp. increased between raw wastewater and treated wastewater. This differs from what was reported by Numberger D. et al., where they found a decrease in the abundance of these two genera (9.5% to 1.3%) between raw wastewater and treated wastewater [48].

The increase in the abundance of *Enterococcus* spp. and *Staphylococcus* spp. in the treated wastewater was probably related to lower competition, since the abundance of the other bacterial genera decreased (Figure 6), which has been reported in other studies [56]. Another explanation for this increase could be the ability of these genera (*Enterococcus* spp., *Staphylococcus* spp., Acinetobacter spp., and *Pseudomonas* spp.) to form biofilm [57,58,59,60].

In our study, we observed that the bacterial species of the *ESKAPEE* group had a relative abundance depending on the bacterial genus detected, where *E. coli*, *K. pneumoniae*, and *P. aeruginosa* were the most abundant species. This is different from what was reported by Hubeny J. et al. [61], who mention that *A. baumannii* and *E. coli* were the dominant pathogens. The dominant abundance of *Enterobacteriaceae* (*E. coli*, *Klebsiella pneumoniae*) in raw wastewater samples has been mainly associated with human feces [50,62]. Furthermore, some of these selected bacteria can survive the different WWTP processes and persist due to their own characteristics [18].

It has been suggested that wastewater released into the environment is a major source of antibiotic-resistant bacteria (ARB) and ARGs, with healthcare facilities being a major source of ARB, due to the discharge of antibiotic residues, Karkman et al. mention that human and animal gut microbiota contain a wide range of ARGs, so sewage discharge and fecal contamination have been linked to the increased abundance of ARGs in the aquatic environment [14,46]. In this study, it was observed that raw wastewater had an average ARG abundance of 99.3 ± 9.6% and treated wastewater showed an average ARG abundance of 20.0 ± 17.2%, observing a reduction of 70%. Szczepanowski R et al. [63] reported a 13% reduction in ARG abundance in treated wastewater from a WWTP in Germany. On the other hand, Yang Y. et al. [64] and Gupta S. et al. [65] reported that wastewater treatment eliminated >99% of ARGs in urban WTTPs in Hong Kong and South Korea, respectively.

Our results showed the presence and persistence of *bla*_KPC-1_, *bla*_OXA-1_, *bla*_OXA-2_, *bla*_OXA-10_, and *bla*_OXA-232_ genes in raw and treated wastewater. The *bla*_KPC_ and *bla*_OXA_ genes were previously detected by PCR in the isolation of *Klebsiella* spp. collected from these same samples, which correlates with the findings obtained by metagenomics [9]. The genes *bla*_TLA-1_, *bla*_TLA-2_, *bla*_TEM-1_, *bla*_MOX-2_, *bla*_MOX-6_, *bla*_MOX-8_, and *bla*_MOX-9_ were only present in the raw wastewater, while the genes *bla*_GES-1_, *bla*_GES-20_, and *bla*_NDM-1_ were detected in a lower proportion in treated wastewater samples. The presence of these ARGs has been detected in the bacteria of the *ESKAPEE* group in hospital wastewater from Romania [66], as well as in urban wastewater in Canada [67] and other countries [15,46,52,68]. Furthermore, it is essential to highlight the presence of environmental bacteria, which play an important role in the abundance of antibiotic-resistant bacteria in aquatic ecosystems, and which, in these water environments, allow for the persistent release of ARGs [69,70,71].

Although the presence of the *mcr* gene that encodes colistin resistance was not detected in our study, this gene has been significantly detected in urban wastewater in other countries such as Spain [72], France [73], Germany [74], and Tunisia [75].

In this study, the genes *mph*(A-E), *msr*(D), *mef*(B), Inu(b), and *ermB*, encoding resistance to MLS, were mainly detected in raw wastewater and persisted in treated wastewater, which agrees with the results of Pallares-Vega. R. et al., who detected these same genes in the wastewater of 62 WWTPs studied in the Netherlands [76], with the *ermB* gene being among the most abundant and persistent. This gene was originally detected in Gram-positive bacteria (*Enterococcus* spp., *Staphylococcus* spp., and *Streptococcus* spp.), but could be transferred to Gram-negative bacteria via a conjugative transposon [18,75].

Furthermore, this study detected the presence of the genes *strA*, *aph(3*″), *aads*, *aadA2*, and *aac3*,which encode resistance to aminoglycosides. The presence of these genes was significantly more abundant in the raw wastewater analyzed by Raza S. et al. in South Korea [77] and Pärnänen K. et al. in the European Union [18].

A study published by Berglund F. et al. in 2023 [51] reported that the most abundant ARGs were associated with bacteria from the *ESKAPEE* group, representing 70% of the total ARGs detected in the samples analyzed; these mainly encode *β*-lactams, aminoglycosides, and macrolides. These results were similar to those observed in our work, where the highest abundance of ARGs was found to encode these three classes of antibiotic families. The less abundant ARGs were probably not detected in the analyzed samples due to the temporality, number of samples, and low sensitivity of the metagenomic tools.

The pattern of ARGs present in hospital wastewater samples detected by metagenomics could be related to the use of antibiotics prescribed in these hospitals. This is supported by the results of a previous study carried out by the working group, where it was reported that third-generation cephalosporins and carbapenems were the most used antibiotics [78], suggesting that the prescription of antibiotics in the hospital setting influences the microbiome and resistome present in wastewater.

Similar results were observed in a study carried out in 12 hospital WWTPs in the European Union (2022) [18], which found that raw wastewater from hospitals with a high consumption of antibiotics had a significantly higher relative abundance of ARGs compared to hospitals with a low consumption of antibiotics. The study observed that these ARGs decreased after wastewater treatment. If this phenomenon continues (antibiotic prescription–presence of ARGs in wastewater), the analysis of wastewater in other hospitals in Mexico would probably have similar results to those found in this work [79].

The present work has some limitations. The study was based on a cross-sectional design, where two hospitals were sampled. The first hospital (HRAEI) had three replications and the second hospital (INCAN) had only two. Other hospitals were not included due to restrictions due to the COVID-19 pandemic. However, the hospital wastewater analysis allowed us to detect the abundance of bacteria from the *ESKAPEE* and ARG group as a first approach to the context in our country.

## 5. Conclusions

Despite the limited number of samples analyzed, our results showed the presence and persistence of *ESKAPEE* group bacteria in hospital wastewater, as well as the presence of ARGs that code for resistance to antibiotics of clinical importance. This pilot study could serve as a model for the early surveillance of AMR in hospital wastewater in our country. Antimicrobial resistance cannot simply be addressed within healthcare facilities; it must also be addressed beyond the facilities of its waste.

## Data Availability

The reads are available from NCBI SRA under BioProject ID PRJNA1010860.

## Supplementary Materials

The following supporting information can be downloaded at: https://www.mdpi.com/article/10.3390/microorganisms12061231/s1, Table S1: Quality control; Table S2: Resistance genes, plasmids, and virulence factors that persist between raw wastewater and treated wastewater.
