# Supplementary material for: Presence and Persistence of ESKAPEE Bacteria before and after Hospital Wastewater Treatment
Source: Microorganisms. 2024 Jun 19;12(6):1231. doi: 10.3390/microorganisms12061231 (PMC11206169; doi:10.3390/microorganisms12061231)
Supplement: Supplementary file 1 [file microorganisms-12-01231-s001.zip › Supplement Table S2.pdf]

Supplement S2. Resistance genes, plasmids, and virulence factors that persist from raw wastewater to treated wastewater**.**

GEN	PLASMIDS	VIRULENCE FACTORS
bla_{OXA}	ColKP3-1	*icmJ*
bla_{VEB}	IncQ2_1	*dotN*
bla_{KPC}	ColRNAI_1	*htpB*
bla_{GES}	Col440I_1	*icmW*
sul	Col440II_1	*fliG*
mph		*flhA*
mef		*flgC*
erm		*fleQ*
msr		*fleN*
ant(3'')		*algC*
aads		*pilT*
lnu		*flhD*
PBP-2		*cheY*
dfra		
van_{A-G}		
tet		
